# Factors associated with atypical radiological findings of pulmonary tuberculosis

**DOI:** 10.1371/journal.pone.0220346

**Published:** 2019-07-25

**Authors:** Akihiko Goto, Kosaku Komiya, Takamasa Kan, Kokoro Honjo, Sonoe Uchida, Shuichi Takikawa, Tetsuyuki Yoshimatsu, Kiminori Fujimoto, Takeshi Johkoh, Jun-ichi Kadota

**Affiliations:** 1 Internal Medicine, National Hospital Organization Nishi-Beppu Hospital, Tsurumi, Beppu, Oita, Japan; 2 Respiratory Medicine and Infectious Diseases, Oita University Faculty of Medicine, Idaigaoka, Hasama-machi, Yufu, Oita, Japan; 3 Radiology, Kurume University School of Medicine, 67-Asahimachi, Kurume, Japan; 4 Radiology, Kansai Rosai Hospital, Inabasou, Amagasaki, Hyogo, Japan; The Foundation for Medical Research, INDIA

## Abstract

**Background:**

Unusual radiological images may delay diagnosis of pulmonary tuberculosis. This study aimed to analyze the risk factors for an atypical radiological image in patients with pulmonary tuberculosis.

**Methods:**

We retrospectively analyzed data from patients admitted to one hospital from January 2013 to December 2016 for sputum smear-positive lung tuberculosis who underwent chest computed tomography (CT) on admission. Patients whose sputum cultures were positive for general bacteria were excluded. Patient characteristics and laboratory data were compared between patients with cavity and those without and between patients with upper predominant lung involvement and those without.

**Results:**

This study included 94 (93%) of 101 patients who underwent chest CT. The non-cavity group was older, had a greater number of females, had a lower C-reactive protein (CRP) level, and had a lower glomerular filtration rate. Multivariate analysis showed that a low CRP level (OR 0.808; 95% CI 0.674–0.967; p = 0.020) significantly predicted non-cavity pulmonary tuberculosis. The non-upper predominant lung involvement group was older and had a greater number of females, poorer performance status, a higher CRP level, and a lower serum albumin level. A poor performance status (OR 2.155; 95% CI 1.257–3.693; p = 0.005) was found to significantly predict pulmonary tuberculosis with non-upper predominant lung distributions.

**Conclusions:**

A low CRP level and poor performance status were associated with non-cavity and non-upper predominant lung distribution, respectively, in patients with pulmonary tuberculosis. Tuberculosis patients with these characteristics may present unusual chest images.

## Introduction

A delayed diagnosis of tuberculosis (TB) can lead to a more advanced disease state as well as can increase the transmission rate of TB infection from one person to another. The causes of the delay in diagnosis include patient’s delay, i.e., the interval between the onset of TB-related symptoms and first visit to a doctor, and doctor’s delay, i.e., the interval between a patient’s first visit to the doctor and TB diagnosis. The factors associated with each source of delay have been studied, and the presentation of an unusual chest image, such as non-cavity disease or non-upper predominant lung involvement, was found to be a risk factor for doctor’s delay [1].

Non-cavity chest images are typically seen among patients with immunosuppressed status, such as human immunodeficiency virus (HIV) infection, advanced age, or immunosuppressant drug use [2, 3]. This status may decrease the lymphocyte and macrophage functions recruited to create granulomatous lesions and be followed by cavitation [4]. Upper lung lobes and segment 6 are typically affected in secondary TB as an endogenous reactivation of past infection that can be explained by higher oxygen tension and lower blood flow, i.e., a higher ventilation/perfusion (V/Q) ratio than the base lung fields [5, 6]. In fact, posterior predominant lung involvement is commonly seen in quadrupedal animals with pulmonary TB, and base predominant lung lesions are seen in bats with pulmonary TB [7]. In elderly people confined to bed who typically rest in the supine position, the V/Q ratio would be higher in the ventral lung field. The ventral lung field may thus be affected in such individuals.

Previous studies have assessed the relationship between underlying diseases and non-cavity disease or unusual lung distributions in pulmonary TB [8]. However, there has been no study that analyzed the risk factors for unusual radiographic presentation that accounted for confounding factors such as physical activity levels. If such predictors were known, doctor’s delay could be reduced by screening the chest image more carefully in consideration of the risk factors. This study thus aimed to identify predictors of non-cavity disease and non-upper predominant distributions among pulmonary TB patients.

## Methods

### Patients

This was a retrospective observational study conducted at National Hospital Organization Nishi-Beppu Hospital, the only official institute with the capacity to accept patients with smear-positive lung TB in Oita Prefecture, Japan. The necessary sample size was determined to be 138 patients, calculated as per previous report [9].

This study included consecutive patients admitted to the hospital between January 2013 and December 2016 for smear-positive pulmonary TB who underwent chest CT within 2 weeks before or after admission. Patients whose sputum culture was positive for general bacteria were excluded because co-infection may modify the chest features. The study protocol was approved by the Institutional Ethics Committee of National Hospital Organization Nishi-Beppu Hospital, Oita, Japan (Approval Number: 30–18; Approval Date: 31 January 2019). Informed consent was waived by the committee because of the retrospective design. Some of the subjects included in this study had already participated in previous report [10].

### Data collection

Patient data—including the gender, age, body mass index (BMI), daily physical activity levels and underlying diseases (e.g. chronic obstructive pulmonary disease, cardiac diseases and diabetes mellitus), laboratory data (e.g. white blood cell count, C-reactive protein [CRP] level, albumin [Alb] level, alanine transaminase and estimated glomerular filtration rate [eGFR]), presence of respiratory failure, and chest CT findings—were obtained from clinical records. The collection of this patient information and examination performance are routinely recommended when a patient diagnosed with lung TB is admitted to our hospital. We evaluated daily physical activity on admission using a scale of performance status (PS). PS was defined by the Eastern Cooperative Oncology Group (ECOG) as follows: 0: fully active, able to carry out all pre-disease activities without restriction; 1: restricted in physically strenuous activity, but ambulatory and able to carry out light and sedentary work; 2: ambulatory and capable of all self-care, but unable to carry out any work activities, up and about more than 50% of waking hours; 3: capable of only limited self-care, confined to a bed or a chair more than 50% of waking hours; and 4: completely disabled, cannot carry out any self-care, totally confined to a bed or a chair [11]. The definition of respiratory failure was below 90% SpO_2_ without oxygen inhalation on admission. The data were extracted by respiratory physicians (AG, TK, KH, SU, ST and KK).

### Evaluation of chest CT findings

A 16-detector rows CT scanner (Activion, Toshiba Medical Systems, Tokyo, Japan) was used at the study hospital. Scans were obtained using 1.0-mm thick sections of contiguous images from the apex to the lung base. Images were photographed at a window setting of –600 HU (level) and 1600 HU (width). If the patient underwent CT before referral to our hospital, we evaluated the CT features from the images taken at the referring institutes.

Two respiratory medicine specialists (with 16 and 10 years of experience), who were blinded to all clinical information, retrospectively assessed the presence of cavity on chest CT and the distribution of any lung involvement. Investigators determined the presence of the features on each 10 and 8 segments in the right and left lungs. The presence of lung involvement was defined as occupation of more than 50% in each lung segment. Upper predominant lung distribution was defined as the presence of lung involvement in right upper lobe, apicoposterior (S1+2) and/or anterior segments (S3) of left upper lobe, and/or superior segments (S6) of bilateral lower lobes segments and the absence of other segments. Middle predominant lung distributions were defined as the presence of lung involvement in right middle lobe and/or lingular segments of left upper lobe (S4 and/or 5) and the absence of other segments. Lower predominant lung distributions were defined as the presence of lung involvement in basal segments of bilateral lower lobes (S7, 8, 9, and/or 10) and the absence of other segments. Cases in which patients did not correspond to the above-mentioned definitions were defined as diffuse distribution.

### Statistical analyses

Statistical analyses were performed using the IBM SPSS statistics ver. 24 software package (IBM SPSS, Tokyo, Japan). Interobserver agreement was assessed by the kappa value. For two-tailed analyses, the confidence intervals (CIs) were 95%. Logistic regression analysis was used for patients with atypical radiological images after adjusting for other variables. Statistical significance was defined as a p value <0.05 for all analyses.

Variables with a p value <0.05 in univariate analysis were then entered into multivariate analysis. As Alb level can reflect both nutrition and inflammation level, BMI and CRP level were included in the multivariate analysis to clarify the individual influence if both factors had p values <0.1 in the univariate analysis.

## Results

### Patient recruitment

**Fig 1:**
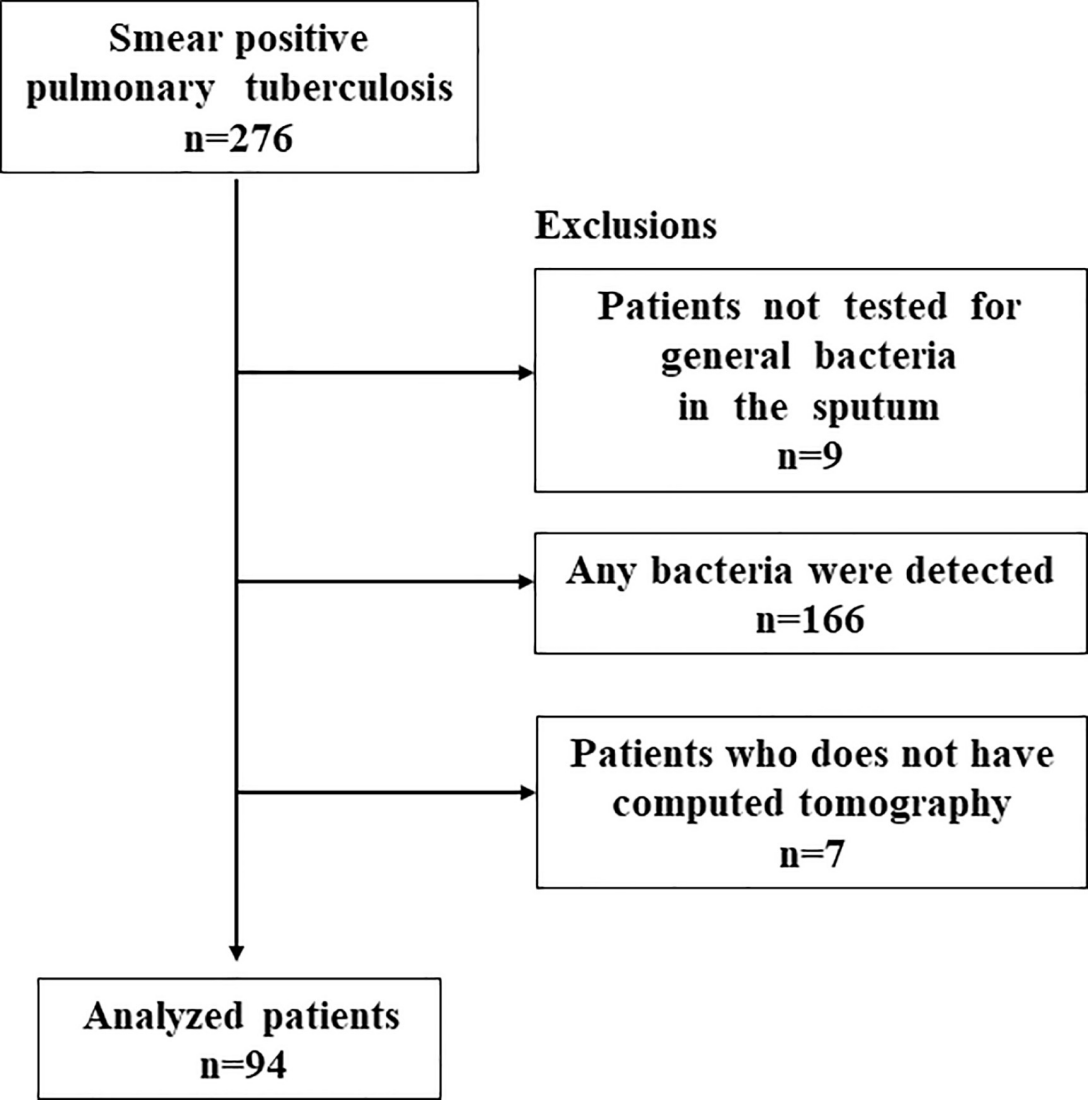
Flowchart of participant selection.

In total, 276 patients with smear-positive TB were admitted to the hospital during the study period. Among them, 267 (97%) underwent general sputum examinations; 166 patients with isolated general bacteria in their sputum culture were excluded. Of the remainder, 7 of 101 did not undergo CT scan within 2 weeks before or after admission, and so, they were excluded. This ultimately resulted in a sample of 94 patients, as shown in Fig 1. The kappa values of the CT findings between the two respiratory medicine specialists were 0.91 for cavity and 0.87 for pattern of lung involvement distribution, respectively.

### Risk factors for non-cavity disease

Non-cavity disease was found in 45 (48%) of 94 patients. The non-cavity group was significantly older and had a greater number of female, a lower CRP level, and a lower glomerular filtration rate (Table 1). Multivariate analysis showed that only low CRP level was significantly associated with non-cavity disease after adjustment for other variables (Table 2).

**Table 1:** Univariate logistic regression analysis of baseline characteristics associated with non-cavity disease.

	Non-cavity (n = 45)	Cavity (n = 49)	Crude odds ratio	p value
Gender (female)	29 (64)	18 (37)	3.122 (1.344–7.249)	0.008
Age (years)	83 (76–88)	76 (62–82)	1.025 (1.000–1.050)	0.048
BMI (kg/m^2^)	20.0 (18.0–21.4)	19.6 (17.9–21.5)	1.040 (0.916–1.181)	0.546
Performance Status 0	5 (11)	2 (4.1)	1.016 (0.725–1.424)	0.928
1	12 (27)	22 (45)		
2	11 (24)	5 (10)		
3	11 (24)	14 (29)		
4	6 (13)	6 (12)		
WBC (/μl)	6600 (5100–7700)	7100 (6100–9700)	0.903 (0.787–1.036)	0.146
Lymphocyte (/μl)	1114 (765–1530)	878 (570–1318)	1.000 (1.000–1.001)	0.194
CRP (mg/dl)	1.4 (0.3–2.3)	2.7 (0.7–4.9)	0.828 (0.711–0.965)	0.016
Alb (g/dl)	3.3 (2.9–3.9)	3.0 (2.5–3.5)	1.524 (0.830–2.796)	0.174
ALT (IU)	15 (11–20)	16 (11–25)	0.995 (0.971–1.020)	0.715
eGFR (mL/min/1.73m^2^)	67.9 (49.3–84.1)	82.9 (65.2–97.9)	0.981 (0.965–0.997)	0.023
COPD	1 (2.2)	3 (6.1)	0.348 (0.035–3.478)	0.369
Cardiac diseases	9 (20)	5 (10)	2.200 (0.677–7.150)	0.190
Diabetes Mellitus	7 (16)	11 (22)	0.636 (0.223–1.817)	0.398
Respiratory failure	4 (8.9)	7 (14)	0.585 (0.159–2.151)	0.420

Data are presented as the number (%) or median (interquartile range).

Alb: albumin, ALT: alanine transaminase, BMI: body mass index, COPD: chronic obstructive pulmonary disease, CRP: C-reactive protein, eGFR: estimate glomerular filtration rate, WBC: white blood cell

**Table 2:** Multivariate logistic regression analysis of baseline characteristics associated with non-cavity disease.

	adjusted odds ratio	p value
Gender (female)	2.304 (0.915–5.802)	0.077
Age (years)	1.018 (0.990–1.047)	0.213
CRP (mg/dl)	0.808 (0.674–0.967)	0.020
eGFR (mL/min/1.73m^2^)	0.989 (0.968–1.010)	0.307

CRP: C-reactive protein, eGFR: estimate glomerular filtration rate

### Risk factors for non-upper predominant disease

**Fig 2:**
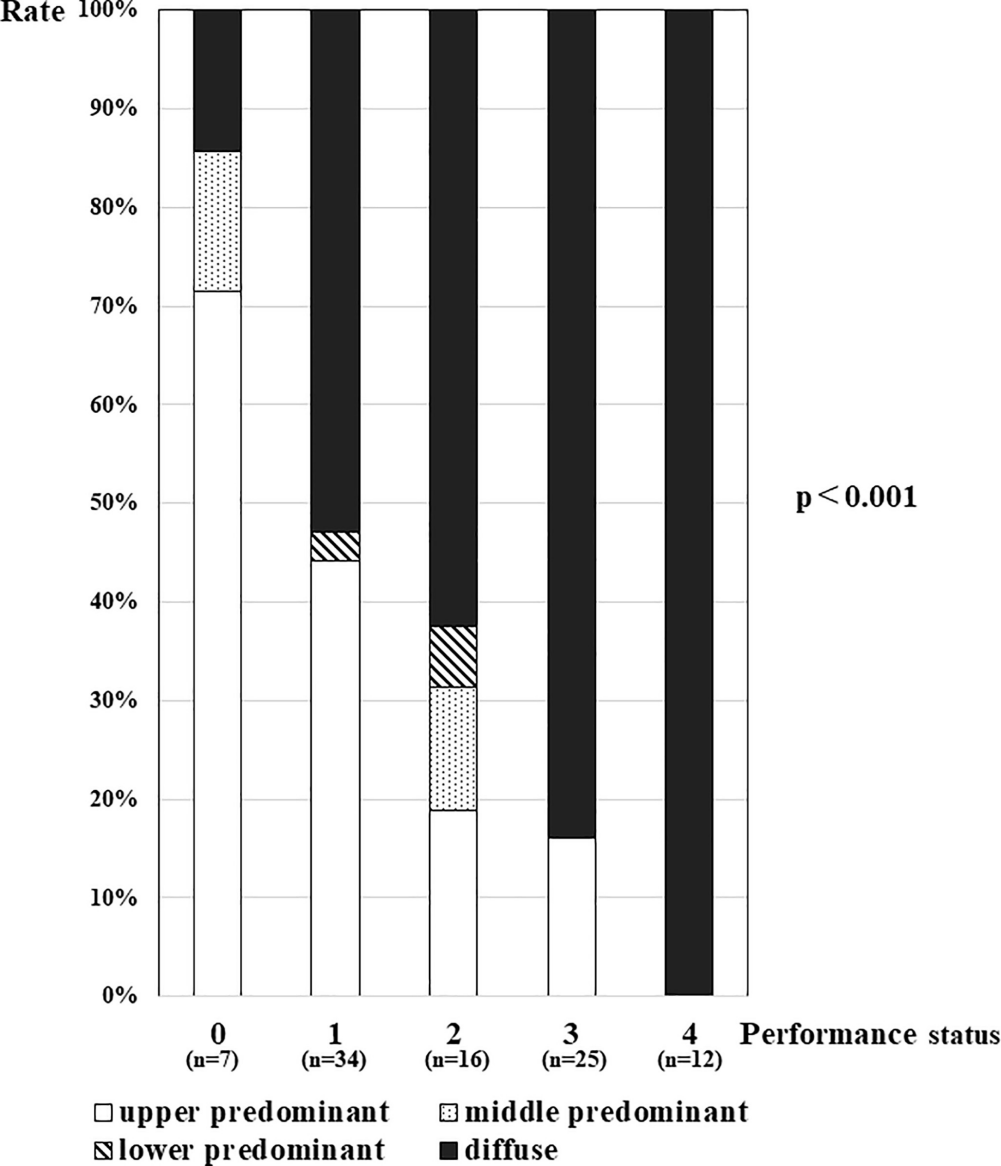
The relationship between performance status and lung distribution in patients with pulmonary tuberculosis. Upper predominant distributions were seen in 71% in PS0, 44% in PS1, 19% in PS2, 16% in PS3, and 0% in PS4; middle predominant distributions were seen in 14% in PS0, 0% in PS1, 13% in PS2, 0% in PS3, and 0% in PS4; lower predominant distributions were seen in 0% in PS0, 2.9% in PS1, 6.3% in PS2, 0% in PS3, and 0% in PS4; and diffuse distributions were seen in 14% in PS0, 53% in PS1, 63% in PS2, 84% in PS3, and 100% in PS4.

Non-upper predominant lung distribution was found in 67 (71%) of 94 patients. The non-upper predominant lung involvement group had a greater number of female with poorer PS, a higher CRP level, and a lower serum Alb level (Table 3). Multivariate analysis showed that poor PS significantly predicted non-upper predominant lung distribution (Table 4). Patients with poorer PS presented a higher rate of diffuse distribution, and all patients with the poorest PS (PS4) presented diffuse distribution (Fig 2). Middle or lower predominant distributions were less frequently observed in each PS level group compared to the upper predominant or diffuse lung distribution.

**Table 3:** Univariate logistic regression analysis of baseline characteristics associated with non-upper predominant lung distribution.

	Non-upper predominant lung distribution (n = 67)	Upper predominant lung distribution (n = 27)	Crude odds ratio	p value
Gender (female)	38 (57)	9 (33)	2.621 (1.029–6.674)	0.043
Age (years)	82 (74–87)	72 (61–80)	1.020 (0.997–1.043)	0.096
BMI (kg/m^2^)	19.5 (17.7–21.2)	20.1 (19.6–21.6)	0.887 (0.771–1.022)	0.097
Performance Status 0	2 (3)	5 (19)	2.601 (1.563–4.329)	<0.001
1	19 (28)	15 (56)		
2	13 (19)	3 (11)		
3	21 (31)	4 (15)		
4	12 (18)	0 (0)		
WBC (/μl)	6900 (5900–8750)	6900 (4850–8500)	1.084 (0.926–1.269)	0.317
Lymphocyte (/μl)	897 (568–1474)	1139 (937–1592)	0.999 (0.998–1.000)	0.070
CRP (mg/dl)	2.6 (1.0–4.7)	0.7 (0.1–2.2)	1.421 (1.083–1.863)	0.011
Alb (g/dl)	3.0 (2.5–3.5)	3.5 (3.2–4.0)	0.216 (0.089–0.522)	0.001
ALT (IU)	16 (10–23)	15 (13–23)	1.015 (0.980–1.051)	0.414
eGFR (mL/min/1.73m^2^)	72.9 (49.0–92.3)	82.8 (67.9–89.0)	0.988 (0.971–1.006)	0.183
COPD	1 (1.5)	3 (11)	0.121 (0.012–1.222)	0.073
Cardiac diseases	13 (19)	1 (3.7)	6.259 (0.776–50.457)	0.085
Diabetes Mellitus	16 (24)	2 (7.4)	3.922 (0.836–18.401)	0.083
Respiratory failure	11 (16)	0 (0)	n.d.	0.999
Cavity	34 (51)	15 (56)	0.824 (0.336–2.022)	0.673

Data are presented as the number (%) or median (interquartile range).

Alb: albumin, ALT: alanine transaminase, BMI: body mass index, COPD: chronic obstructive pulmonary disease, CRP: C-reactive protein, eGFR: estimate glomerular filtration rate, n.d.: not detected, WBC: white blood cell

**Table 4:** Multivariate logistic regression analysis of baseline characteristics associated with non-upper predominant lung distribution.

	adjusted odds ratio	p value
Gender (female)	2.849 (0.967–8.394)	0.057
BMI (kg/m^2^)	0.930 (0.783–1.105)	0.410
Performance Status	2.155 (1.257–3.693)	0.005
CRP (mg/dl)	1.256 (0.967–1.631)	0.088

BMI: body mass index, CRP: C-reactive protein

## Discussion

This study demonstrated that a low CRP level and poor PS were associated with non-cavity and non-upper predominant lung distribution, respectively, in patients with pulmonary TB.

While the Alb level and eGFR in the non-cavity group were significantly lower than those values in the cavity group in univariate analyses, these variables were not significant after adjusting for the CRP level. The Alb level and eGFR may be confounded by the CRP level as an inflammation marker because inflammation increases capillary permeability and escape of serum albumin, leading to a reduction in the eGFR. CRP is an acute phase protein produced in the liver in response to systemic inflammation [12]. A low CRP level might indicate a mild inflammation status due to the small amount of *M*. *tuberculosis* exposed to cell-mediated immunological lymphocytes. A cavity develops after expectoration of purulent matter from granuloma lesions with significant immune cell recruitment to the sites of TB infection. Thus, in the early phase of TB infection with a low CRP level, granulomatous lesions may be developing, but cavitation might not yet have occurred. Furthermore, the CRP level is not generally elevated in immunocompromized hosts compared to healthy adults when they are suffering from infectious diseases [13]. In immunocompromized hosts with low CRP levels, granuloma lesions and cavitation might not develop even under severe disease conditions.

A meta-analysis comparing the clinical characteristics between elderly and younger adult patients with pulmonary TB showed that non-cavity disease was more common in elderly patients [2]. Advanced age was not found to be a predictor of non-cavity disease in our study likely because the included patients were significantly aged (median age, 79 years), which might attenuate the impact of age on cavity disease presentation. Pérez-Guzman et al. reported that TB patients with diabetes mellitus (DM) had cavity disease more frequently than patients without DM [9]. It is known that DM causes the dysfunction of polymorphonuclear leukocytes and reduces bactericidal activity [14]; thus, cavity development may be more progressive in DM patients with pulmonary TB [15]. However, decreased immunity may also be related to the lower frequency of cavitation in DM patients, as discussed above. It remains controversial whether DM has a positive or negative impact on cavity disease. DM was not found to be a predictive factor of cavity disease in our study. However, most DM in study patients might be controlled because the universal health coverage and checkup system are well-developed in Japan [16]. Although TB patients with HIV infection have the potential to present with non-cavity diseases, only five patients in this study were screened for HIV, and all had negative results. Physicians cannot enforce HIV screening in Japan for ethical reasons. Thus, the rate of HIV infection could be underestimated in our study population.

Non-upper predominant lung distribution was more frequently observed in patients with poor PS after adjustment for other variables such as BMI and CRP level. There are two possible reasons for this finding. First, the V/Q ratio would be higher in the ventral lung field in poor PS patients—especially those with bed-ridden status in the supine position [17]—which could affect the non-upper lung field. Second, patients with poor PS may have swallowing dysfunction and thus have difficulties in expectorating sputum [18]. Patients with decreased swallowing function can present gravity-dependent lower lung involvement in general pneumonia as well as in pulmonary TB [19]. Although advanced age is reported to be a predictive factor for non-upper predominant distribution [20], a significant association between advanced age and distribution was not found in our study. As some elderly people are able to maintain a good level of physical activity, aging and decreased PS are not necessarily correlated. Poor PS, rather than advanced age, appears to have a significant impact on non-upper predominant distribution.

Our study has several strengths. It is the first report to assess the relationship between CRP or PS and radiological features in patients with TB. Although several studies assessing chest X-ray features in pulmonary TB have been published, there have been no such studies assessing chest CT. Chest CT is increasingly used in pulmonary TB to exclude the possibility of other diseases such as malignancy, especially in middle- or high-income countries. Therefore, the results of the CT evaluation in our study will be informative to physicians in this area. Furthermore, it is known that co-infection with general bacteria can modify the lung features; however, previous studies did not consider this issue. Our study excluded patients whose sputum culture was positive for general bacteria to reduce this source of measurement bias as much as possible.

There are also some limitations of our study. First, as this was a single-centered retrospective study that included a large population of elderly patients, the results are not generalizable to countries with younger populations. However, as many countries are expected to face aging and aged society in the near future [21], these findings are still useful. Second, we cannot completely exclude co-infection with general pathogens. Even though we excluded patients whose sputum culture was positive for general bacteria, this method is not perfect, and the results of the sputum culture would have been negative if patients were administered antibacterial agents before transfer to our hospital. Third, we did not consider the disease duration from onset to diagnosis. Patients with a long disease duration before CT may have a progressive disease, and the incidence of cavitation would increase. Unfortunately, we were not able to determine the timing of the disease onset in our dataset because most elderly patients had endogenous reactivation of TB [22].

In conclusion, a low CRP level and poor PS were associated with non-cavity and non-upper predominant lung distribution, respectively, in patients with pulmonary TB. TB patients with these characteristics may present unusual chest images. The findings of this study could be applied in clinical practice to reduce doctor’s delay for TB diagnosis.

## Abbreviations

BMI: Body Mass Index
CI: confidence intervals
CRP: C-reactive protein
CT: Computed tomography
DM: diabetes mellitus
ECOG: Eastern Cooperative Oncology Group
HIV: human immunodeficiency virus
PS: performance status
TB: tuberculosis
V/Q ratio: ventilation/perfusion ratio
